# Na^+^/K^+^-ATPase α1 Identified as an Abundant Protein in the Blood-Labyrinth Barrier That Plays an Essential Role in the Barrier Integrity

**DOI:** 10.1371/journal.pone.0016547

**Published:** 2011-01-31

**Authors:** Yue Yang, Min Dai, Teresa M. Wilson, Irina Omelchenko, John E. Klimek, Phillip A. Wilmarth, Larry L. David, Alfred L. Nuttall, Peter G. Gillespie, Xiaorui Shi

**Affiliations:** 1 Department of Otolaryngology/Head and Neck Surgery, Oregon Hearing Research Center, Oregon Health & Science University, Portland, Oregon, United States of America; 2 Proteomic Shared Resources, Oregon Health & Science University, Portland, Oregon, United States of America; 3 Kresge Hearing Research Institute, University of Michigan, Ann Arbor, Michigan, United States of America; 4 Department of Otolaryngology, Renji Hospital, Shanghai Jiao Tong University, Shanghai, China; 5 The Institute of Microcirculation, Chinese Academy of Medical Sciences and Peking Union Medical College, Beijing, China; 6 Vollum Institute, Oregon Health & Science University, Portland, Oregon, United States of America; Katholieke Universiteit Leuven, Belgium

## Abstract

**Background:**

The endothelial-blood/tissue barrier is critical for maintaining tissue homeostasis. The ear harbors a unique endothelial-blood/tissue barrier which we term “blood-labyrinth-barrier”. This barrier is critical for maintaining inner ear homeostasis. Disruption of the blood-labyrinth-barrier is closely associated with a number of hearing disorders. Many proteins of the blood-brain-barrier and blood-retinal-barrier have been identified, leading to significant advances in understanding their tissue specific functions. In contrast, capillaries in the ear are small in volume and anatomically complex. This presents a challenge for protein analysis studies, which has resulted in limited knowledge of the molecular and functional components of the blood-labyrinth-barrier. In this study, we developed a novel method for isolation of the stria vascularis capillary from CBA/CaJ mouse cochlea and provided the first database of protein components in the blood-labyrinth barrier as well as evidence that the interaction of Na^+^/K^+^-ATPase α1 (ATP1A1) with protein kinase C eta (PKCη) and occludin is one of the mechanisms of loud sound-induced vascular permeability increase.

**Methodology/Principal Findings:**

Using a mass-spectrometry, shotgun-proteomics approach combined with a novel “sandwich-dissociation” method, more than 600 proteins from isolated stria vascularis capillaries were identified from adult CBA/CaJ mouse cochlea. The ion transporter ATP1A1 was the most abundant protein in the blood-labyrinth barrier. Pharmacological inhibition of ATP1A1 activity resulted in hyperphosphorylation of tight junction proteins such as occludin which increased the blood-labyrinth-barrier permeability. PKCη directly interacted with ATP1A1 and was an essential mediator of ATP1A1-initiated occludin phosphorylation. Moreover, this identified signaling pathway was involved in the breakdown of the blood-labyrinth-barrier resulting from loud sound trauma.

**Conclusions/Significance:**

The results presented here provide a novel method for capillary isolation from the inner ear and the first database on protein components in the blood-labyrinth-barrier. Additionally, we found that ATP1A1 interaction with PKCη and occludin was involved in the integrity of the blood-labyrinth-barrier.

## Introduction

The cochlear blood-labyrinth-barrier, located in the stria vascularis, is essential for cochlear solute homeostasis and prevents the influx of toxic substances into the inner ear [1], [2]. Disruption of the blood-labyrinth barrier is closely associated with the pathogenesis of a number of hearing disorders, such as autoimmune inner ear disease, Meniere's disease, meningitis-associated labyrinthitis and genetic diseases [3], [4], [5], [6]. Furthermore, loss of blood-labyrinth barrier integrity occurs early and prominently during noise-induced hearing loss [7]. Development of new treatments for blood-labyrinth barrier disruption-related hearing loss requires a better understanding of the molecular structure of the blood-labyrinth barrier, as well as the molecular mechanisms that control the barrier integrity.

Many proteins of the blood-brain barrier and blood-retinal barrier have been identified, leading to significant advances in our understanding of their tissue specific functions [8], [9]. In contrast, stria vascularis capillaries are small in volume and anatomically complex. This presents a challenge for protein analysis studies, which has resulted in limited knowledge of the molecular and functional components of the blood-labyrinth barrier. However, recent technical advances have allowed for proteomic identification and quantification of blood barrier proteins by advanced analytical mass spectrometry. In this paper, we describe a novel “sandwich-dissociation’’ method for isolation of stria vascularis capillaries. Using a mass spectrometry-based, shotgun-proteomics approach [10], more than 600 proteins were identified from isolated mouse stria vascularis capillaries. Strikingly, a high number of identified proteins were predicted to be involved in the metabolic and transport processes required to meet the high metabolic requirements of audition. For example, the most abundant protein identified in the blood-labyrinth barrier was the ion transporter Na^+^/K^+^-ATPase α1 subunit (ATP1A1). In addition, stria vascularis capillaries were found to be enriched for tight junction (TJ) and cell adhesion proteins that are indicative of the blood-labyrinth barrier's impermeable nature.

Na^+^/K^+^-ATPase, a heterodimer of catalytic α and β subunits, is a membrane bound enzyme primarily involved in generation of Na^+^ and K^+^ gradients across plasma membranes and in determination of cytoplasmic Na^+^ levels [11], [12]. The Na^+^ pump generates this ionic gradient through ATP dependent pumping of three Na^+^ ions out of the cell and two K^+^ ions in. The Na^+^ pump is essential for the maintenance of physiological functions of many cell types by regulating cell volume, intracellular ion balance and tight junction protein [13], [14]. Furthermore, in this study, we found interactions between the transporter, ATP1A1, a protein kinase, PKCη, and a TJ protein, occludin, in the blood-labyrinth barrier, decreased Na^+^/K^+^-ATPase activity results in occludin hyperphosphorylation by PKCη and loosening of TJ contact points causing increased permeability in noise-induced blood-labyrinth barrier disruption.

## Materials and Methods

### Animals

The 130 CBA/CaJ mice used in this study (6 weeks old, both male and female, Cat# 000654) were purchased from Jackson laboratory (Bar Harbor, Maine, USA). All procedures were reviewed and approved by the Institutional Animal Care and Use Committee at Oregon Health & Science University (IACUC approval number: B11265).

### Cochlear stria vascularis capillary isolation

Cochlear stria vascularis capillaries were isolated from 6-week-old CBA/CaJ mice. The capillary isolation was achieved with a special procedure we developed and named the “sandwich-dissociation” method. Specifically, after cardiovascular perfusion with saline, the auditory bulla was dissected, rapidly removed, and placed in a petri dish filled with a physiological solution containing 125 mmol/L NaCl, 3.5 mmol/L KCl, 1.3 mmol/L CaCl2, 1.5 mmol/L MgCl2, 0.51 mmol/L NaH2PO4, 10 mmol/L HEPES, and 5 mmol/L glucose at pH 7.4 with osmolarity adjusted to 310 mmol/kg. Each turn of the bony cochlear lateral wall containing the spiral ligament and stria vascularis was separated from the organ of Corti, and the stria vascularis was gently peeled away from the spiral ligament with Dumont Tweezers (110 mm, 0.1×0.06 mm tip) and a Tungsten Dissecting Probe (50 mm, 0.5 mm diameter rod) under a dissecting microscope (Olympus, SZ61). The stria vascularis was placed in a glass-bottom microwell dish (dish diameter: 35 mm; microwell diameter: 14 mm; coverglass: 0.16–0.19 mm; MatTek Corp.) filled with the physiological solution. A glass coverslip (0.16 mm) was then positioned over the stria vascularis, and the tissue was gently sandwiched between the glass surfaces. Gentle pressure was applied to compress the stria vascularis against the two glass surfaces. By repeating this step, the non-vascular tissues were dispersed into the solution and separated from the capillaries. Non-vascular cells were gently flushed away with 100 µl micropeptide, while the clean microvessels adhered and “printed” onto the bottom of the dish, which is analogous to the offset-printing deposit of a pattern of ink onto paper. The isolated capillaries were then transferred to a microfuge tube. For mass spectrum analysis, capillaries were collected from a total of 100 mice cochlear stria vascularis. After the initial dissection, all the steps were performed under a laminar-flow hood to reduce keratin contamination.

### Protein identification by LC-MS/MS and data analysis

The tryptic peptides were analyzed by 2D-LC-MS/MS. In brief, a 100×2.1 mm strong cation-exchange (SCX) column (Polysulfoethyl A, 5 µm particle size, 300 Å pore diameter) was prepared for ion-exchange chromatography as instructed by the manufacturer (The Nest Group, Inc.). Tryptic peptides were injected onto the SCX column at a 200 µl/min flow rate. The operating conditions for the analytical column were mobile phase A containing 10 mmol/L sodium phosphate (pH 3.0) and 25% acetonitrile, along with mobile phase B containing 10 mmol/L sodium phosphate (pH 3.0), 25% acetonitrile and 350 mmol/L potassium chloride. Following 5 minutes of loading and washing in mobile phase A, peptides were eluted using a linear gradient of 0% to 50% B over 45 min, followed by a linear gradient of 50% to 100% B over 20 min. One-minute fractions were collected and dried by vacuum centrifugation and then redissolved by shaking in 100 µl of 5% formic acid. Portions of each fraction (40 µl) were analyzed by LC-MS/MS using an Agilent 1100 series capillary LC system and an LTQ linear-ion trap mass spectrometer (Thermo Inc.) using a standard electrospray source fitted with a 34-gauge metal needle (Thermo Inc.). Chromatography was achieved using an Eksigent nanoLC to generate a gradient using the following chromatographic conditions: mobile phase A: water, acetonitrile, formic acid, trifluoroacetic acid (95, 4.89, 0.1, 0.01, v/v/v/v); mobile phase B: acetonitrile, isopropanol, water, formic acid, trifluoroacetic acid (80, 10, 9.89, 0.1, 0.01, v/v/v/v/v). Mobile phase B was ramped from 2% to 45% over 40 minutes, increased to 80% in 5 minutes, and held for 5 minutes before being returned to starting conditions. The flow was regulated at 200 nl/min and directed through a 75 µm ×150 mm column packed in house with Astrosil (5 µm particle size, 100 Å pore size, C18 reverse phase chemistry; Stellar Phases) coupled to a 5 µm tapered emitter (New Objectives). Prior to analytical chromatography, 5 µl of tryptic digest was injected onto a 150 µm ×20 mm sample trap packed with Poros R10; the trap was washed with mobile phase A to remove salts and contaminants and then was switched in line with the analytical column. Tandem mass spectrometry data was collected using a QSTAR XL hybrid time-of-flight mass spectrometer (Applied Biosystems) under the following conditions: spray voltage 1800 to 1900 V; TOF-MS scan m/z 400 to 1600, 0.5 sec; TOF-MS/MS scan m/z 50 to 2000, 2.0 sec, 9 sec exclusion.

LC-MS/MS data were converted to DTA files using extract_msn software (Thermo Inc.) with the following parameters: MW range 550 to 4000, group scan 1, minimum group count 1, minimum ion count 25, and an absolute threshold of 500. The 175,961 DTA files were analyzed using SEQUEST (version27, Thermo Inc.) and a suite of in-house software [15]. A mouse species subset of the Swiss-Prot protein database (version57.2; 16,115 entries) was prepared with concatenated reversed entries and searched with SEQUEST. A parent ion mass tolerance of 2.5 Da was used with average parent ion mass and average fragment ion masses. Cys had a static modification mass of +57 Da (alkylation with iodoacetimide). No enzyme specificity was used. Discriminant function thresholds were set independently by peptide charge, and the number of tryptic termini was set to allow 34,982 identifications, where 445 matches were to reversed entries for an estimated peptide false discovery rate of 1.3%. Protein identification lists were prepared using DTASelect (Version 1.9, the Scripps Research Institute) with post processing of results to improve spectral-count accuracy and allow more strict protein identification criteria. Proteins were considered present in a given sample, if they had two or more peptides with distinct sequences, having a unique count greater than or equal to one in the respective sample. The number of proteins (including contaminants) was 652, with 9 matches to reversed entries for an estimated protein false discovery rate of 1.3%. Each protein identification was assigned a cellular function by PIPE (http://pipe.systemsbiology.net/pipe/#Summary) based on Genome Ontology (GO) terms.

### Noise exposure

Animals were exposed to broadband noise at 120 dB SPL for three hours for two consecutive days. This noise-exposure protocol is routinely used in our laboratory and produces a permanent cochlear-sensitivity loss [16].

### Protein determination

In this study, total protein in each different experiment was measured in a fluorescence microplate reader using with a CBQCA protein quantitation kit (Cat# C-6667, Invitrogen). Isolated stria vascularis capillaries from one mouse were mixed with 140 µl 0.1 mol/L borate buffer (pH 9.3), sonicated for 2 min, and incubated for 30 min on ice to allow protein extraction. After a short centrifugation at 12,000 rpm, 135 µl of the capillary extract was sampled for protein quantitation. The plates were incubated for 1 h at room temperature, and the increase in fluorescence was assayed at emission (535 nm) and at excitation (485 nm) using a TECAN GENios Plus ELISA reader (TECAN) and the XFluo 4 software (TECAN). Bovine serum albumin was used as the quantitation standard. Control experiments were carried out to ensure that the borate buffer did not interfere with protein quantitation.

### Immunofluorescence confocal microscopy

The tissue samples were fixed in 4% paraformaldehyde at 4°C for 2 h, washed in PBS for 30 min, permeabilized in 0.5% Triton X-100, then immunoblocked in a solution of 10% goat serum in 1% bovine albumin in PBS for 30 min. The specimens were incubated with primary antibodies (see antibodies below) and secondary antibodies (goat Alexa Fluo 488-conjugated anti-mouse and anti-rabbit IgG antibodies, Invitrogen). The fluorescence was visualized on an Olympus IX81 inverted microscope fitted with an Olympus Fluoview FV1000 confocal laser microscope system. The samples were examined as above, and Z-series stacks were examined at an interval of 1 µm. The Z-series images were projected using Image J 1.30 software (National Institutes of Health) and processed by Adobe Photoshop (vCS3, Adobe Systems).

### Transmission electron microscopy and scanning electron microscopy

For the transmission electron microscopy, cochlear lateral wall tissue was dissected from the control and noise-exposed animals. Segments of the cochlear lateral wall from the basal turns were fixed overnight in phosphate-buffered 3% glutaraldehyde-1.5% paraformaldehyde and post-fixed in 1% osmium. The tissues were dehydrated, embedded in Araldite plastic, sectioned, stained with lead citrate and uranyl acetate, and viewed in a Phillips, CM, 100 transmission electron microscope (Eindhoven, the Netherlands).

For the scanning electron microscopy, isolated capillary samples were fixed and imaged by using an ESEM (FEI Quanta 200, Hillsboro, OR) at a stage height of approximately 10 mm, 15 kV, and magnification of 4000×. The recorded images were saved in TIFF format.

### Immunoblots

The total lysate from the stria vascularis capillaries was obtained by sonicating the tissue in RIPA lysis buffer (Cat# 87787, Thermo Inc.) with a protease inhibitor mixture (Cat# P8340, Calbiochem). After centrifugation, the supernatant was collected, and the protein was quantified by the CBQCA assay described above. Protein extracts were separated by SDS/PAGE and analyzed by immunoblots with relevant antibodies. For densitometry, non-saturated developed films were scanned using a Canon LiDE scanner (Canon, USA), and mean pixel density of each band wasmeasured using Image J 1.30 software (National Institutes of Health). Measurements were standardized to actin loading control, and fold changes vs. control was calculated.

### Co-immunoprecipitation assay

Interaction between ATP1A1 and occludin or PKCη was determined by coimmunoprecipitation. Equal amounts of protein from the stria vascularis capillary lysates prepared with RIPA buffer were incubated on a rotator overnight with an antibody bound to protein A agarose beads (Cat# sc-2001, Santa Cruz Biotechnology, Inc) at 4°C. The proteins bound to the beads were separated by SDS-PAGE, and coimmunoprecipitating proteins were analyzed by immunoblotting.

### Protein-protein interaction assay

Purified recombinant ATP1A1 protein (250 ng) and purified recombinant PKCη protein (250 ng) were incubated together in 250 µl PBS at 4°C for 2 h. Next, either anti- ATP1A1 antibody or anti-PKCη antibody was incubated with the reactions for 4 h at 4°C. Where indicated, ouabain (10 µmol/L) was added followed by additional 1 h incubation. Protein A/G agarose beads (Santa Cruz Biotechnology, Inc.) were then added to the reactions and incubated overnight at 4°C with rocking. The beads were washed four times with PBS and boiled in a Laemmli sample buffer. The samples were separated by SDS-PAGE, and the proteins were detected by immunoblotting with indicated antibodies.

### RT-PCR

Total RNA from the cochlear stria vascularis capillaries was separately extracted, with RNeasy (QIAGEN), from the control and NE groups. Each group was comprised of five mice for analysis of mRNA levels of Na^+^/K^+^-ATPase α1 (ATP1A1), β1 (ATP1B1) and β2 (ATP1B2) with quantitative RT-PCR. Two µg of total RNA and 100 ng of random hexamer were used to make 20 µl of cDNA by SuperScript II (Invitrogen) following the manufacturer's instructions. The cDNA synthesized from total RNA was diluted 10-fold with DNase-free water. Transcript quantities were assayed by corresponding TaqMan gene expression assay: ATP1A1 (Cat# Mm00523255_m1; Applied Biosystems), ATPB1 (Cat# Mm00437612_m1) and ATP1B2 (Cat# Mm00442612_m1) with a model 7300 real-time PCR system (Applied Biosystems). Thermal cycle conditions were an initial hold of 50°C for 2 m, 95°C for 10 m, then 40 cycles of 95°C for 15 s and 60°C for 1 m. The samples were run in triplicate for each gene. Quantitative PCR was performed according to the guidelines provided by Applied Biosystems. The comparative cycle threshold (C*_T_*) method (ΔΔC*_T_* quantitation) was used to calculate the difference between samples. Quantitative data analysis was followed according to the suggestions of the manufacturer.

### Enzymatic activity assay

Both PKCη and Na^+^/K^+^-ATPase activity were respectively determined with a PKCη KinEASE^TM^ FP Fluorescein Green Assay Kit (Millipore) and an ATPase assay kit (Novus Biologicals). For the PKCη activity measurement, the stria vascularis capillary homogenates were mixed in the reaction buffer, which was composed of KinEASE buffer and ATP. The PKCη activity was assayed using a Fluoresence Polarization Reader (BioTek) with excitation at 458 nm and emission at 530 nm. For the Na^+^./K^+^-ATPase activity measurement, the isolated stria vascularis capillary homogenates were mixed in the reaction buffer which consisted of 50 mmol/L Tris, 2.5 mmol/L MgCl2 and 0.5 mmol/L ATP. The total ATPase activity was assayed in the reaction mixture. The resting ATPase activity, except Na^+^/K^+^-ATPase, was assayed in the presence of an additional 1 mmol/L ouabain in the reaction mixture. Na^+^/K^+^-ATPase specific activity was calculated by subtracting the resting ATPase activity from the total ATPase activity. The enzyme activity was determined by the amount of inorganic phosphate (Pi) released from the substrate ATP. The Pi concentrations were read using a TECAN GENios ELISA reader (TECAN) at a wavelength of 595 nm. Enzyme specific activity equals concentration (µmol) of Pi liberated per mg protein per hour.

### Permeability analysis

Vascular permeability was assessed using the method based on determining the limits of the stria vasculature capillaries and the diffusion limits of the IgG extravasated around the vessel. As more permeability on the BLB (more extravasations) generates a higher gradient of IgG, and diffusion depends on the gradient, high levels of IgG will extend longer from the vessel. Image treatment and fluorescence analyses of the images were performed by means of the Image J software (National Institutes of Health, USA). In order to determine the limits of the capillaries the strategy consisted on the following steps: First, on the image showing collagen-IV immunostaining, arbitrary lines were traced transversally crossing each blood vessel. Second, the profile intensity of the staining along each line was plotted. Third, a horizontal threshold line defining valid data was drawn over each profile level in order to define the limits of stain positiveness. Fourth, the diameter of each vessel (VD) was determined after converting pixels to µm. In order to determine the limits of IgG extravasation, the last three steps were applied on the equivalent images corresponding to IgG staining, using the same lines transversally crossing each blood vessel. The extension of the extravasation (EE) was obtained by converting pixels of the positively stained IgG region to micrometers (Figure S1).

### Drug application

The PKCηPS peptide, Myr-TRKRQRAMRRRVHQING [17], and a control peptide with scrambled sequence, (PKCηNC) (Myr-RMINKARVRGRAQRHG-OH) [18], were custom synthesized by GenScript. Both the peptides and ouabain solution were prepared by being dissolved in a physiological solution. For *in vivo* studies, sterile surgical procedures were used to apply 10 µl of a 0.5 mmol/L PKCηPS solution to the round window (RW) for 30 m prior to applying 1 mmol/L ouabain (Cat# 4995, Calbiochem) to the RW or exposing animals to noise. The PKCηNC solution treated ears served as a control. For *in vitro* studies, the isolated stria vascularis capillaries were treated with 50 µmol/L of PKCηPS solution for 30 m before treating with 100 µmol/L ouabain for 5 hrs. The isolated capillaries treated with PKCηNC solution served as control samples for the experiments.

### Antibodies and purified proteins

The following antibodies were used in this study: mouse monoclonal anti-actin (Cat# MAB1501, Chemicon), anti- Na^+^/K^+^-ATPase α1 (Cat# 05-369, Millipore), anti-desmin (Cat# ab6322, Abcam), anti-PKCη (Cat# 610814, BD Transduction); rabbit polyclonal anti-Kir 4.1(Cat# AB5818, Chemicon), anti-occludin (Cat# 40-4700, Invitrogen), anti-ZO-1 (Cat# 40-2300, Invitrogen), anti-Collagen type IV (Cat# ab6586, Abcam), anti-NG2 (Cat# NBP1-20170, Novus Biologicals), anti-PECAM-1(Cat# ab24590, Abcam). The purified recombinant proteins used in this study were: ATP1A1 (Cat# TP301009) from Origene Technologies, Inc. and PKCη (Cat# 10-782-55103) from Geneway Biotech, Inc.

### Statistics

Data are presented as mean ± SEM of at least three independent experiments with three or more replicates and analyzed by wilcoxon signed-rank test, with P<0.05 considered significant.

## Results

### Isolation of stria vascularis capillaries

**Figure 1:**
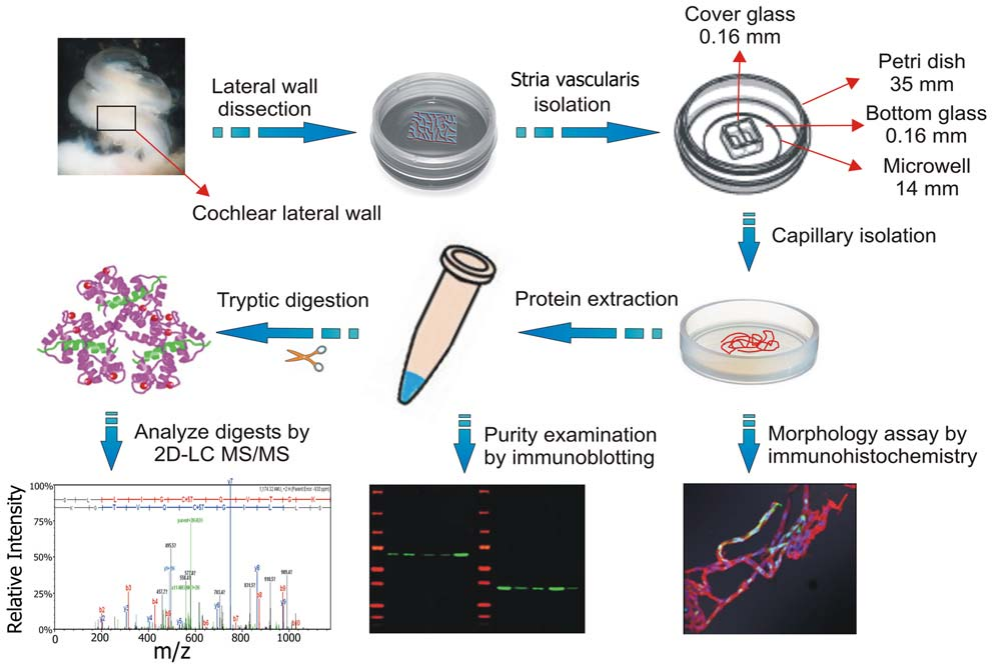
Isolation of capillaries from the stria vascularis of adult mouse cochlea. Flow diagram of the steps involved in capillary isolation from the stria vascularis and downstream processing for immunohistochemistry, immunoblot, and 2D-LC MS/MS analysis.

**Figure 2:**
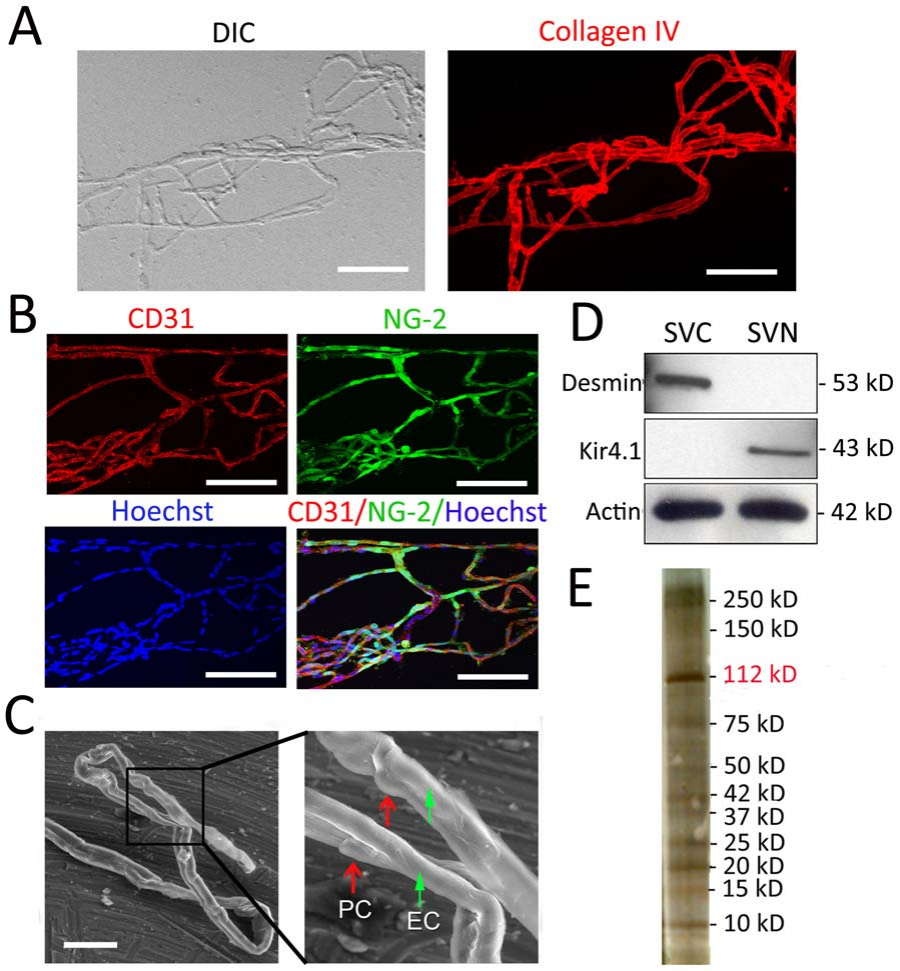
Characterization of isolated SV capillaries. **A**, A DIC image (Left) of an isolated mouse SV capillary network obtained by the “sandwich-dissociation” method (see *Materials and Methods*). The capillary is immunolabeled with an antibody for collagen type IV to show the basement membrane (Right). **B**, Images of isolated capillaries triple labeled for EC marker protein CD31 (red channel), PC marker protein NG-2 (green channel), and Hoechst for nuclei (blue channel) verify absence of non-vascular cell contamination in the capillary preparation. **C**, A scanning electron micrograph of isolated capillaries (Left), with the accompanying higher magnification image, shows PCs on the isolated capillaries (Right, arrows). **D**, Immunoblot analysis of the isolated capillaries. The pericyte marker protein desmin is detected only in the capillary fraction, while the intermediate cell marker protein Kir 4.1 is only detected in the non-vascular fraction, confirming the preparation is uncontaminated by intermediate cells. SVC, stria vascularis capillary fraction; SVN, stria vascularis non-vascular fraction. **E**, One-dimensional SDS-PAGE separation of the isolated capillary proteins, silver stained. Migration positions corresponding to the NKA α1 subunit (112 kD) and its major bands are indicated (Scale bars: **A** and **B**, 100 µm; **C**, 50 µm) Data in all panels are representative of at least 3 separate experiments.

In this study, a unique sandwich-dissociation method was developed to isolate stria vascularis capillaries from the mouse cochlea. The procedure is illustrated in Figure 1 (for detailed information see *Materials and Methods*). Preparation purity was assessed by differential interference contrast (DIC), confocal fluorescence microscopy and scanning electron microscopy (SEM). Representative images are shown in Figure 2. Isolated capillaries labeled for collagen type IV, a predominant component of basement membrane, define the capillary structure, as shown in Figure 2A. Additional immunolabeling with CD31, an endothelial cell (EC) marker protein, and NG-2, a pericyte (PC) marker protein, showed the cellular composition of the isolated capillaries (Figure 2B). Hoechst staining detected cell nuclei only in vascular cells, including PC and EC (Figure 2B), indicating the absence of non-vascular cell contamination. SEM verified the lack of non-vascular cells contamination (Figure 2C).

Immunoblot analysis for known protein markers of PCs and intermediate cells (IC) further verified preparation purity. ICs, non-vascular cells, are a likely contaminate as these cells form a honeycomb network and surround capillaries with their dendritic processes [19]. While Kir 4.1, an IC marker, was not detected in the capillary fraction indicating the preparation was free of ICs, desmin, a PC marker protein, was detected in the capillary fraction (Figure 2D). Next, capillary protein extracts were separated by SDS-PAGE, and a protein migrating with the same molecular weight as ATP1A1 (112 kD) was predominant in the purified capillaries (Figure 2E), consistent with the proteomic data described below. Although minimal contamination cannot be ruled out for individual preparations, we are confident our isolation was sufficiently pure to achieve a high degree of blood-labyrinth barrier protein enrichment.

### Categorization of stria vascularis capillary proteins identified ATP1A1 subunit as the most abundant protein in the blood-labyrinth barrier

Capillary protein (19 µg) obtained from 100 cochleae was digested with trypsin and analyzed by 2D-LC/MS/MS. A total of 652 proteins were identified with an estimated false discovery rate of less than 1.5%. Details of the analysis and a list of identified proteins are found in Dataset S1. The relative mass-abundance of proteins in shotgun proteomic samples is proportional to the number of assigned MS/MS spectral counts [20]. While the accuracy of spectral counting for estimation of relative protein abundance has not been rigorously tested, the technique provides a useful ranking of protein abundance in complex mixtures. The identified capillary proteins were ordinally listed by assigned MS/MS spectra (Dataset S1).

**Figure 3:**
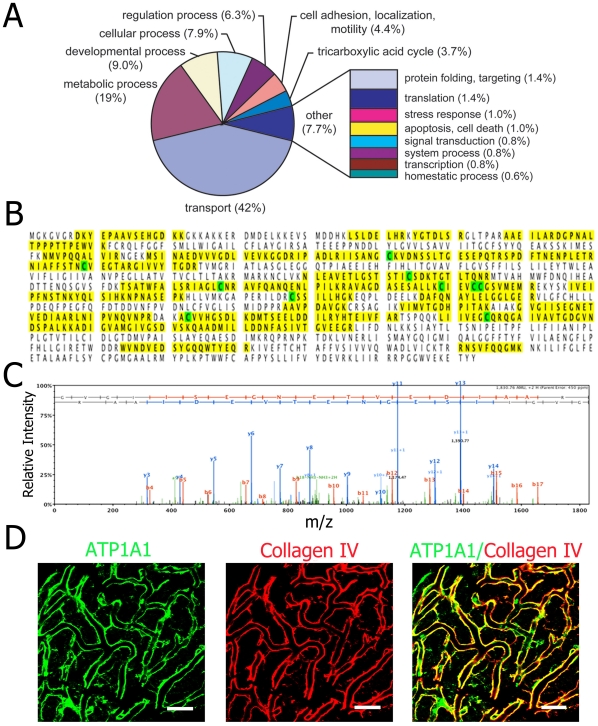
Classification of isolated stria vascularis capillary proteins identified ATP1A1 as the most abundant protein in the blood-labyrinth barrier. **A**, Spectral count-weighted tabulation of GO annotation by biological process. Proteins involved in transport (42%) and metabolism (19%) are highly expressed in the blood-labyrinth barrier. **B**, The sequence of ATP1A1 covered by identified peptides is outlined in yellow (45% coverage). The alkylated residues (green) were identified by iodoacetamide treatment. **C**, A representative MS/MS spectrum for peptide 630–645 of ATP1A1. Graphically displayed are the fragment ions identified by SEQUEST from the tandem mass spectrometry data. Mass-to-charge (m/z) values are indicated next to each peak, along with positions of y-ions (blue) and b-ions (red). Unmatched ions are displayed as black. **D, Localization of ATP1A1 (green) in stria vascularis capillaries (red, antibody for collagen type IV). A merged image (Right) shows the distribution of ATP1A1 along the capillary. Data in all panels are representative of at least 3 separate experiments. (Scale bars: **D**, 20 µm).**

The putative barrier proteome was further analyzed against tabulated categories of a gene ontology database to obtain a functional overview of the blood-labyrinth barrier proteins (Dataset S2). Proteins were classified into categories based on “biological process,” with the categories weighted by total spectral count (Figure 3A). Abundance-weighting of annotations provides an informative picture of tissue function. Annotation analysis was also done for “cellular component” (Figure S2) and “molecular function” (Figure S3). As expected under “biological process,” the blood-labyrinth barrier is rich in proteins for molecular transport (42% of total spectral counts) and metabolic processes (19% of total spectral counts) (Figure 3A). Metabolic enzymes are also highly expressed in the blood-labyrinth barrier, including glutathione S-transferase (GST), prosaposin, leukotriene A4 hydrolase, and glutamate oxaloacetate transaminase. In particular, on a mass basis, ATP1A1 was the most abundant protein; peptides covering 45% of the amino acid sequence of ATP1A1 were identified (Figure 3B). A representative tandem mass spectrum of a ATP1A1 peptide is shown (Figure 3C). Expression of ATP1A1 in stria vascularis capillaries was further validated by immunocytochemistry (Figure 3D).

### ATP1A1 regulation of TJ permeability involves occludin phosphorylation in the blood-labyrinth barrier

**Figure 4:**
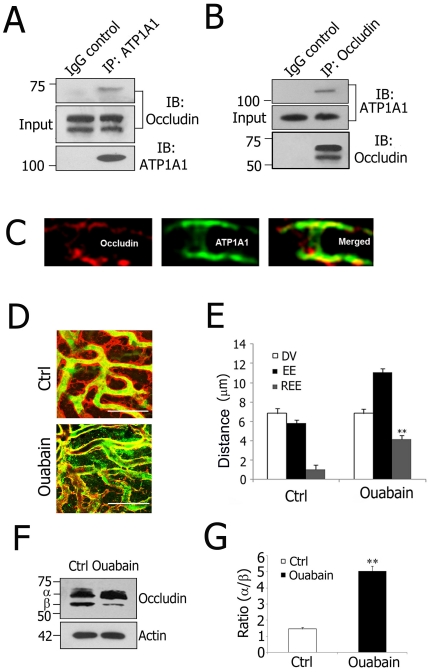
ATP1A1 regulation of TJ permeability involves occludin phosphorylation in the blood-labyrinth barrier. **A** and **B**, Co-immunoprecipitation using isolated stria vascularis capillary lysates demonstrates that ATP1A1 is in a complex with occludin. Preimmune goat IgG served as a negative control. **C**, Immunolabeling of stria vascularis capillaries for occludin (Left, red) and ATP1A1 (Middle, green). The merged image (Right) shows that occludin and ATP1A1 co-localization. **D**, Confocal images show increased TJ permeability in ouabain-treated tissues (Lower) compared to control tissues (Upper). Serum protein IgG (green, antibody for IgG (H+L) is outside the lumen of the capillary (red, antibody for collagen type IV). **E**, REE analysis confirmed the significantly increased permeability of ouabain-treated tissues (**P = 0.005<0.01). **F**, Immunoblot analysis shows occludin migrates as two bands, hyperphosphorylated α (∼70 kD) and dephosphorylated β (∼65 kD), in both control and ouabain-treated tissues. **G**, The ratio of α band to β band in ouabain-treated tissues was significantly increased (**P = 0.007<0.01). Values in **E** and **G** are mean ± SEM (n = 10). (Scale bars: **D**, 20 µm). REE: relative extension of serum protein IgG extravasation; DV: diameter of the vessels; EE: extension of IgG extravasation.

While ATP1A1 is known to regulate TJ permeability through interaction with TJ proteins in the epithelial barrier [21], the present study addresses the question of whether ATP1A1 is necessary for TJ function in the endothelial barrier. The link between ATP1A1 and occludin was investigated by co-immunoprecipitation using capillary protein lysate, with the finding that ATP1A1 is in a complex with occludin (Figure 4A and 4B). By dual-labeling immunofluorescence analysis, ATP1A1 was further identified to co-localize with occludin in the blood-labyrinth barrier (Figure 4C).To determine whether ATP1A1 regulates TJ permeability, ouabain, a specific pharmacological inhibitor of Na^+^/K^+^-ATPase activity [22], was applied to the round window membrane of the cochlea. Blood-labyrinth barrier permeability was then assessed from the relative extension of serum protein IgG extravasation (REE) outward from the capillary wall. Under control conditions, IgG was restricted to the lumen of capillaries (Figure 4D). In contrast, ouabain treatment resulted in a statistically significant increase in blood-labyrinth barrier permeability as demonstrated by leakage of IgG from the capillary wall (Figure 4D and 4E, P<0.01).

To investigate whether the observed change in permeability was associated with TJ reorganization, we examined the effect of ouabain treatment on post-translational modification of a pivotal components of endothelial TJs, occluding. Phosphorylation of occludin is essential for TJ assembly and is a critical regulator of barrier integrity [23], [24], [25]. Isolated stria vascularis capillaries were treated with ouabain and the phosphorylation state of occludin was examined by immunoblot analysis. In the untreated control, occludin migrated as two major bands which included a slower migrating ∼70 kD hyperphosphorylated form (α) and a faster migrating ∼65 kD dephosphorylated form (β) (Figure 4F). Following inhibition of Na^+^/K^+^-ATPase, the α/β ratio was significantly increased, indicating phosphorylation of occludin (Figure 4G). This result suggests that normal Na^+^/K^+^-ATPase activity is required for maintaining the phosphorylation state of occludin.

### Decreased Na^+^/K^+^-ATPase activity increases TJ permeability via PKCη phosphorylated occludin

**Figure 5:**
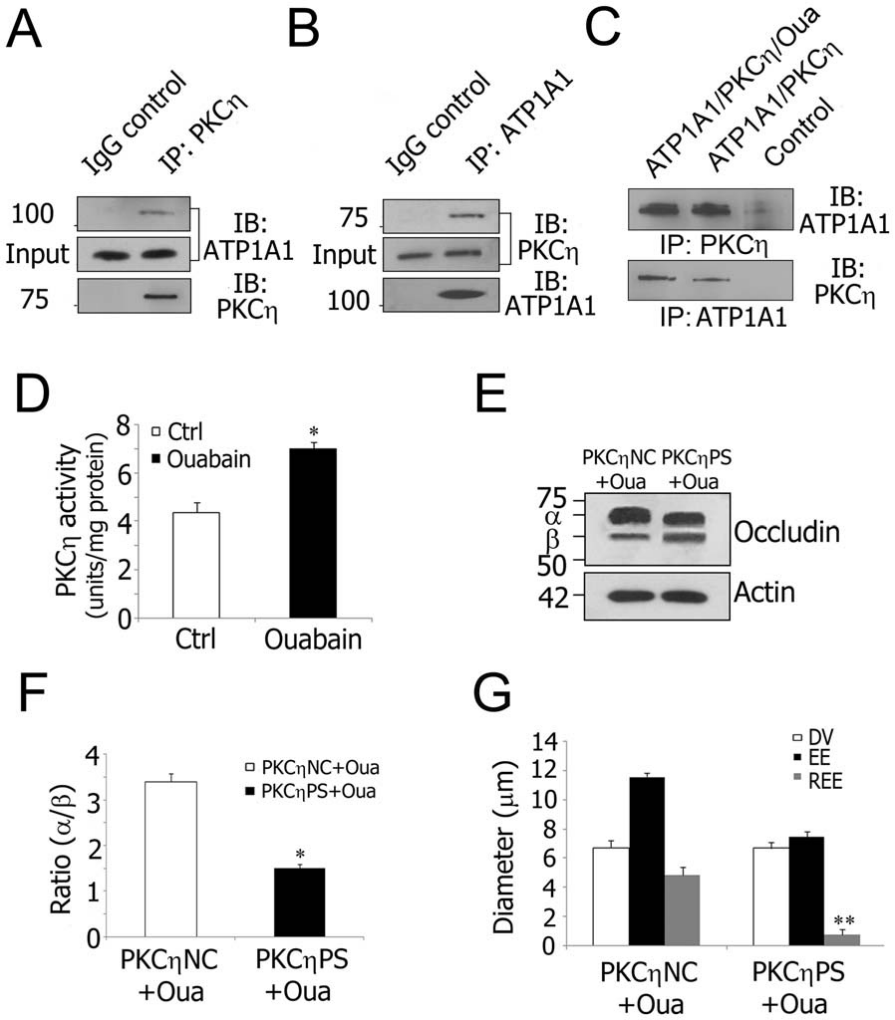
Decreased Na^**+**^/K^**+**^-ATPase activity increases TJ permeability via PKCη phosphorylated occludin. **A** and **B**, Co-immunoprecipitation using isolated stria vascularis capillary lysates shows that PKCη is in a complex with ATP1A1. Goat IgG served as a negative control. **C**, Protein-protein interaction analysis with purified ATP1A1 (250 ng) and PKCη (250 ng). Ouabain (10 µM) was added where indicated. Control lanes consisted of either anti-ATP1A1 antibody and purified PKCη or anti-PKCη antibody and purified ATP1A1. **D**, Ouabain inhibition of Na^+^/K^+^-ATPase activity causes increased PKCη activity in isolated stria vascularis capillaries (*P = 0.013<0.05). **E** and **F**, PKCηPS pretreatment significantly attenuated ouabain-induced occludin hyperphosphorylation compared to pretreatment with PKCηNC (* P = 0.011<0.05). **G**, REE analysis shows a significant attenuation in ouabain-induced permeability in PKCηPS pre-treated tissues (**P = 0.005<0.01). REE, relative extension of serum protein IgG extravasation; DV, diameter of the vessels; EE, extension of IgG extravasation. Values in **D**, **F** and **G** are mean ± SEM (n = 10).

The phosphorylation status of TJ proteins is mediated by several different protein kinases [26] and phosphatases [27]. Of interest, PKCη regulation of epithelial TJ permeability is affected through occludin phosphorylation [18]. To determine whether ATP1A1 induced hyper-phosphorylation of occludin in endothelial TJs could be mediated by PKCη, co-immunoprecipitation using capillary protein lysate was performed. The results provide clear evidence that PKCη and ATP1A1 are in a complex *in vivo* (Figure 5A and 5B). A direct interaction between ATP1A1 and PKCη was shown by an *in vitro* binding assay using purified proteins (Figure 5C). The addition of ouabain (10 µM) to preformed ATP1A1-PKCη complexes did not disrupt the interaction. Next, the functional interaction between ATP1A1 and PKCη was tested. Using a fluorescein assay, total PKCη activity was measured as 4.37 ± 0.43 units/mg protein in control isolated stria vascularis capillaries, which increased significantly to 7.01 ± 0.28 units/mg protein when the capillaries were treated with ouabain for 3 h (Figure 5D). These results suggest that the physical interaction between PKCη and ATP1A1 has functional relevance in regulation of TJ permeability.

To explore this possibility, the interaction between PKCη and occludin phosphorylation was examined. Prior to ouabain treatment, tissues were pretreated with a PKCη pseudo substrate (PKCηPS) which mimics the substrate of PKCη and competitively inhibits PKCη kinase activity [17]. Inhibition of PKCη significantly attenuated ouabain-induced hyperphosphorylation of occludin (Figure 5E and 5F). Reduction of occludin phosphorylation by PKCη activity inhibition resulted in decreased TJ permeability following inhibition of Na^+^/K^+^-ATPase by ouabain (Figure 5G). In contrast, no change was detected in occludin phosphorylation or TJ permeability in tissues pretreated with the control peptide, PKCηNC (Figure 5E–G). The results provide evidence that decreased Na^+^/K^+^-ATPase activity increases endothelial TJ permeability via PKCη phosphorylation of occludin.

### ATP1A1 is implicated in noise-induced disruption of the blood-labyrinth barrier

**Figure 6:**
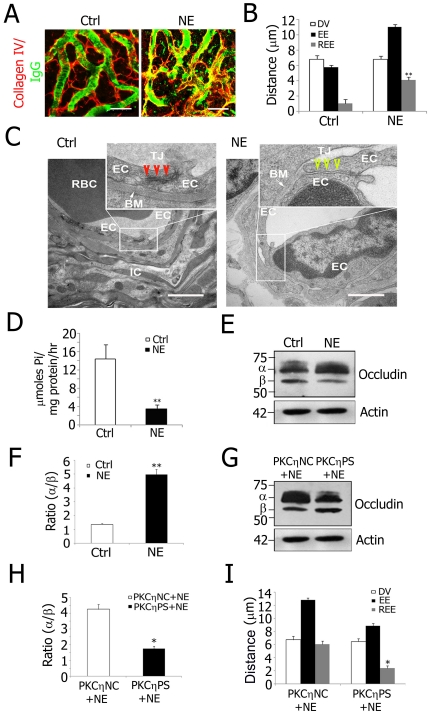
PKCη phosphorylated occludin from decreased Na^**+**^/K^**+**^-ATPase activity is a mechanism for noise-induced blood-labyrinth barrier disruption. A, Confocal images of IgG leakage from capillaries in disrupted blood-labyrinth barrier of noise-exposed (NE) animals. Stria vascularis capillary networks were labeled with anti-collagen type IV (red) and anti-serum protein IgG (H+L, green) antibodies. IgG was confined in normal stria vascularis capillaries (Left) but not in NE animals (Right). B, REE analysis shows significantly increased blood-labyrinth barrier permeability in NE mice (**P = 0.005<0.01). REE: relative extension of serum protein IgG extravasation; DV: diameter of the vessels; EE: extension of IgG extravasation. C, Transmission electron microscope images show alterations in TJ ultrastructure from NE. TJ contact points were reduced in response to NE (Right, Inset). In contrast, TJ contact points were extensive in control mice (Left, Inset). Insets display higher magnification of TJ between ECs. D, NE caused decreased Na^+^/K^+^-ATPase activity in stria vascularis capillaries in NE animals (** P = 0.005<0.01). E, Immunoblots for occludin from isolated stria vascularis capillaries of control and NE mice. Two bands, hyperphosphorylated α (∼70 kD) and dephosphorylated β (∼65 kD), were detected. F, Densitometry of the immunoblot results found a 3.9-fold increase in the (α/β) ratio following NE (**P = 0.005<0.01). G, Immunoblots for occludin from NE mice pre-treated with PKCηNC and PKCηPS. H, A 2.4-fold decrease in the (α/β) ratio occurred with PKCηPS pre-treatment (*P = 0.022<0.05). I, REE analysis confirms decreased permeability in NE mice pre-treated with PKCηPS (*P = 0.021<0.05). Values in B, D, F, H, and I are mean ± SEM (n = 10). BM: basal membrane; EC: endothelial cell; IC: intermediate cell; RBC: red blood cell; TJ: tight junction. (Scale bars: A, 20 µm; C, 500 nm).

Noise trauma results in depletion of intracellular ATP as well as in changes in vascular permeability [28]. The link between ATP depletion and permeability changes is the decreased Na^+^/K^+^-ATPase activity that ATP depletion engenders [29]. To assess whether decreased Na^+^/K^+^-ATPase activity, and corollary TJ reorganization, can account for noise-induced vascular leakage, animals were exposed to wide-band noise at 120 dB SPL for 3 h a day for 2 consecutive days. Hearing thresholds at all test frequencies were elevated as assessed by auditory brain-stem response (ABR) (Figure S4). Immediately after noise exposure, blood-labyrinth barrier permeability was visualized with an REE of the serum protein IgG. Confocal imaging showed widespread blood-labyrinth barrier breakdown, with IgG leaking from the stria vascularis capillary wall in noise-exposed (NE) animals (Figure 6A). Quantification analysis revealed a significant increase in blood-labyrinth barrier permeability in the NE animals compared to unexposed control animals (Figure 6B, P<0.01). Further, transmission electron microscope (TEM) imaging displayed a reduced number of TJ contact points in the NE animals in comparison to the control mice (Figure 6C), indicating that loud sound-induced permeability changes are associated with altered TJ organization. Next, Na^+^/K^+^-ATPase specific activity was examined by microcolorimetric assay in isolated stria vascularis capillaries of the control and NE animals, and a remarkable decrease in Na^+^/K^+^-ATPase activity in the NE animals was observed (Figure 6D, P<0.01). Interestingly, expression of ATP1A1 was significantly increased at both the mRNA and protein levels following noise exposure (Figure S5).

Next, investigation was done to determine if post-translational modifications of TJ proteins occurrs upon noise exposure by immunoblot analysis (Figure 6E). The α/β ratio of occludin was significantly increased in the NE animals in comparison to the control mice (Figure 6F, 3.9 fold, P<0.01). The shift to the slower migrating form of occludin can be attributed to a change in phosphorylation status in response to noise exposure. To determine whether noise-exposure leads to PKCη phosphorylation of occludin, PKCηPS was applied to the round window membrane of the cochlea before noise exposure. Inhibition of PKCη activity significantly attenuated both occludin hyperphosphorylation (Figure 6G and 6H), and increased TJ permeability was observed in the untreated NE animals (Figure 6I). No attenuation was found in animals pretreated with the control peptide PKCηNC (Figure 6G–I). The correlation between blood-labyrinth barrier disruptions and the phosphorylation state of endothelial occludin strongly suggests that noise-induced blood-labyrinth barrier leakage is regulated by PKCη activity.

## Discussion

Proteomic screening of the blood-labyrinth barrier using a “sandwich-dissociation” method, in combination with shotgun proteomics, delineated 652 proteins from isolated strial capillaries. ATP1A1, a key component of tight junctions in polarized epithelial cells [30], was identified as the most abundant protein in the barrier. We show here that ATP1A1 regulates TJ permeability in the blood-labyrinth barrier through a mechanism involving occludin phosphorylation.

The blood-labyrinth barrier in the stria vascularis, comprised of a dense capillary network of endothelial cells, surrounding pericytes, and basement membrane [19], is anatomically different from blood barriers in brain and retina. In contrast to the high degree of vascularization and loose association with surrounding nonvascular tissue present in other barriers, the blood-labyrinth barrier is a sandwich of epithelial marginal cells and mesodermal basal cells interconnected by TJs. Dendrite-like projections of intermediate cells, dispersed between the two cell layers, make close contacts with capillaries through gap junctions. The complexity of the capillary beds has made isolation of stria vascularis cells from surrounding non-vascular cells technically challenging. The “sandwich-dissociation” method, reported here, cleanly separates intact stria vascularis capillaries from nonvascular cells (Fig. 2), and enables functional proteomic study of the blood-labyrinth barrier without significant contamination.

The mass spectrometry data was searched against a mouse species subset of the current Swiss-Prot protein database, a comprehensive database of 16,115 entries (Dataset S1). We note, however, the list is not complete. Some TJ proteins are scarce or have a limited number of trypsin cleavage sites, with the effect of underrepresentation. Also, while vascular perfusion removes contaminating cells, blood proteins, such as albumin, are commonly found in proteomic screens of endothelial cells [31]. Despite these caveats, validation experiments indicate most of the proteins in Dataset S1 are authentic blood-labyrinth barrier proteins.

While ATP1A1 is expressed at lower levels in non-vascular tissue [32], our finding of ATP1A1 the most abundant protein in the blood-labyrinth barrier is in concurrence with other studies showing high expression of the subunit in blood-brain barrier [33]. ATP1A1 is a component of the Na^+^/K^+^-ATPase ion transporter. Na^+^/K^+^-ATPase is a ubiquitously expressed oligomeric protein composed of two essential polypeptide subunits, the catalytic α-subunit (∼112 kDa) and the β-subunit (∼55 kDa) [34], [35]. There are four distinct α-subunit isoforms in mammalian cells (α1, α2, α3 and α4). All α-subunit isoforms have 10 transmembrane segments with 5 extracellular loops, intracellular termini, and binding sites for Na^+^, K^+^, ATP, and cardiotonic steroids, such as the specific inhibitor ouabain [36]. Each, however, also has unique kinetic properties and a distinctive response to second messenger signaling [37]. ATP1A1 is considered to be a regulatory molecule, as it plays a central role, not only in cellular ion homeostasis, but also in regulation of permeability, modifying TJ proteins in epithelial barriers of cell lines [30], [38]. In contrast, the Na^+^/K^+^-ATPase β-subunit is associated with the transmembrane helices and may have a role in cell-to-cell adhesion [11].

The phosphorylation state of TJ proteins has a direct bearing on barrier integrity [25]. Treatment with ouabain, a specific inhibitor of Na^+^/K^+^-ATPase [39], increases blood-labyrinth barrier permeability by increasing phosphorylation of occludin (Figure 4D–G). A number of regulators of protein phosphorylation, including protein kinase C [26], tyrosine kinase c-Yes [40], and protein phosphatase PP2A [22], have been reported to be involved in this process.

This study is the first to have shown ATP1A1 is in a protein complex with occludin and PKCη in the endothelial blood-labyrinth barrier (Figure 4A and 4B and Figure 5A and 5B). PKCη is demonstrated to regulate occludin phosphorylation and TJ permeability in epithelia [18], and blockage of PKCη attenuates occludin hyperphosphorylation. The current study, however, provides direct evidence of a functional interaction between ATP1A1 and PKCη. ATP1A1 inhibition upregulates PKCη kinase activity, and subsequent hyperphosphorylation of occludin reduces TJ contact points and increases permeability of the barrier. The mechanism by which ATP1A1 inhibition upregulates PKCη kinase activity was examined by *in vitro* binding analysis, which further confirmed direct interaction between ATP1A1 and PKCη. Moreover, this complex was not disrupted by addition of ouabain to the preformed complexes (Figure 5C). The ouabain-induced conformational change in ATP1A1 likely activates PKCη kinase activity. This finding is analogous to the Na^+^/K^+^-ATPase-Src complex in which ouabain binding by ATP1A1 results in release and activation of the Src kinase domain without disrupting the complex [41].

Acoustic trauma from loud sound causes both mechanical and metabolic damage to cochlear cells [42], [43]. Direct mechanical damage to hair cells is the primary cause of noise-induced hearing loss [44], [45], [46], while disruption of the cochlear blood flow and blood-labyrinth barrier (CBLB) in the stria vascularis further accelerates damage to hair cells by creating an ischemic and hypoxic environment [16], [47], [48]. In particular, noise causes cochlear hypoxia by generating edema in the stria vascularis with breach of the blood-labyrinth barrier [49], while the molecular mechanism for noise induced disruption of the barrier is not fully understood. Mechanical damage and the harmful effects of cytokine mediators, including vascular endothelial growth factor and free radicals [50], [51], [52], [53], have been implicated in the increased vascular permeability with noise exposure [16].

The present study demonstrated for the first time the molecular effect of noise exposure. It showed Na^+^/K^+^-ATPase activity is reduced with noise exposure, leading to PKCη activation and occludin hyperphosphorylation, resulting in vascular leakage and disruption of the blood-labyrinth barrier (Figure 6). Transmission electron microscopy provided corroborating evidence of significantly reduced TJ “contact points” in noise-exposed animals. The mechanism by which noise exposure decreases Na^+^/K^+^-ATPase activity is thought to be through depletion of regional ATP or through direct affects on the structure of the enzyme [54]. Impaired energy metabolism, accompanied by rapid depletion of intracellular ATP, has been reported in noise-exposed animals [28]. As ATP is the prime substrate of the Na+/K+ pump, reduced ATP levels may have the effect of depressing Na^+^/K^+^-ATPase activity, leading to PKCη activation and occludin hyperphosphorylation. Na^+^ would accumulate under conditions of attenuated Na^+^/K^+^-ATPase activity. Interestingly, accumulation of intracellular Na^+^ upregulates genetic expression of ATP1A1. Similarly, upregulated ATP1A1 expression is reported in ouabain-treated rat kidney epithelial cells [55], offseting the enzyme inhibition. We observed significantly increased expression of ATP1A1 (but not the β subunit) at both the transcriptional and translational levels in the strial blood-labyrinth barrier after noise exposure (Figure S5).

In summary, the present study has established the use of proteomic techniques on isolated stria vascularis capillaries. For the first time, global proteomic analysis of the blood-labyrinth barrier revealed a protein component in the barrier. Furthermore, we found that ATP1A1 interaction with PKCη and occludin was involved in the integrity of the blood-labyrinth barrier.

## Supporting Information

Figure S1 **Image analysis strategy designed to quantify the diameter of stria vascualris microvessels and the extent of IgG extravasation.** **A**, Capillaries stained with an anti-collagen IV antibody with an arbitrary (a, b) line traced. **B**, A horizontal threshold is traced, and the vessel diameter (DV) is obtained. **C**, The same vessel stained with anti-IgG antibody and with the same arbitrary line (a, b) traced. **D**, The extension of IgG extravasation (EE) has been determined after drawing the background horizontal line. REE is defined as EE subtracted from DV (REE  =  EE-DV). REE: relative extension of serum protein IgG extravasation; DV: diameter of the vessels; EE: extension of IgG extravasation.
(TIF)

Figure S2 **Isolated capillary protein classification according to cellular component.** **A**, GO annotation by cellular component. 544 proteins were identified to be associated with a diverse cellular component and mitochondrial proteins, including matrix proteins (17%), inner membrane proteins (16%) and outer membrane proteins (1%), were highly expressed in the blood-labyrinth barrier. **B**, Allocation of isolated stria vascularis capillary proteins by cellular component showed the greatest number of these proteins (95) were allocated to a mitochondrial matrix.
(TIF)

Figure S3 **Isolated capillary protein classification according to molecular function.** **A**, GO annotation by molecular function. 526 proteins were identified to be related to molecular functions and proteins involved in catalytic activity (31%) were highly expressed in the blood-labyrinth barrier. **B**, Allocation of isolated stria vascularis capillary proteins by molecular function showed that the greatest number of these proteins (164) was allocated to catalytic activity and the smallest (1) to electron carrier activity.
(TIF)

Figure S4 **ABR hearing thresholds were measured from the noise-exposed and control mice (n  =  10) at 4, 8, 12, 16, 24 and 32 kHz.** *Significantly different from control mice (***P < 0.001). Error bars represent SEM.
(TIF)

Figure S5 **The expression of ATP1A1 in response to noise exposure.** **A**, Real-time PCR showed the expression of mRNAs of Na^+^, ATP1A1, ATP1B1 and ATP1B2 in the control and noise-exposed (NE) animals. ATP1A1 mRNA was highly up-regulated in response to noise exposure. (** P  =  0.0043< 0.01, n  =  3). However, ATP1B1 and ATP1B2 mRNA did not reveal a significant difference in NE animals. **B** and **C**, Immunoblots quantified by densitometry shows that expression of ATP1A1 was significantly increased in the NE animals. (* P  =  0.032 < 0.05, n  =  5).
(TIF)

Dataset S1 **Summary of the 652 proteins identified in stria vascularis capillaries.** Common contaminant, sequence-reversed decoys, and proteins with insufficient unique peptide evidence are not listed.
(XLS)

Dataset S2 **Functional classification and annotation of proteins identified in this stria vascularis capillaries proteomic study.**
(XLS)
