# Supplementary figures and images for: Na+/K+-ATPase α1 Identified as an Abundant Protein in the Blood-Labyrinth Barrier That Plays an Essential Role in the Barrier Integrity
Source: PLoS One. 2011 Jan 31;6(1):e16547. doi: 10.1371/journal.pone.0016547 (PMC3031570; doi:10.1371/journal.pone.0016547)

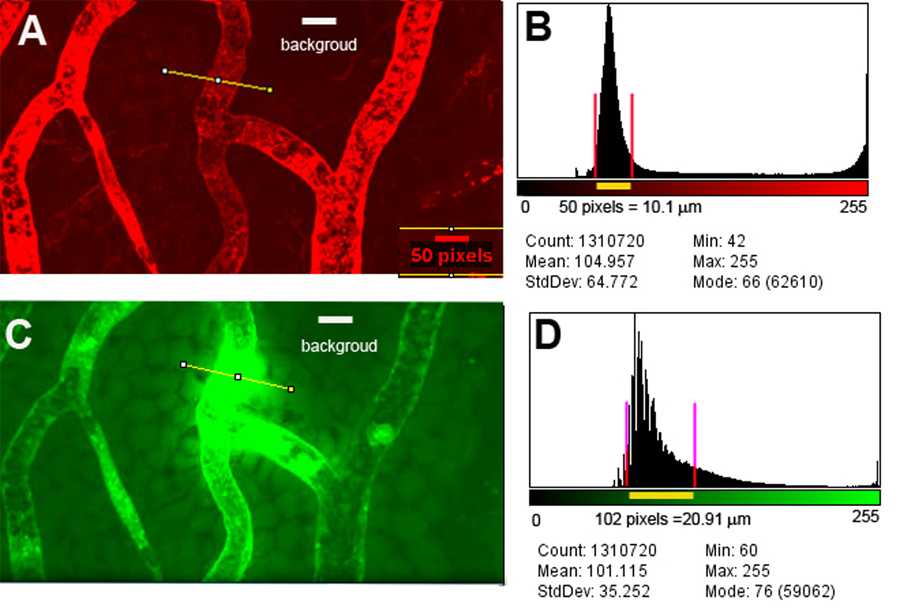

Supplement: Figure S1 — Image analysis strategy designed to quantify the diameter of stria vascualris microvessels and the extent of IgG extravasation. A, Capillaries stained with an anti-collagen IV antibody with an arbitrary (a, b) line traced. B, A horizontal threshold is traced, and the vessel diameter (DV) is obtained. C, The same vessel stained with anti-IgG antibody and with the same arbitrary line (a, b) traced. D, The extension of IgG extravasation (EE) has been determined after drawing the background horizontal line. REE is defined as EE subtracted from DV (REE = EE-DV). REE: relative extension of serum protein IgG extravasation; DV: diameter of the vessels; EE: extension of IgG extravasation. (TIF) [file pone.0016547.s001.tif]

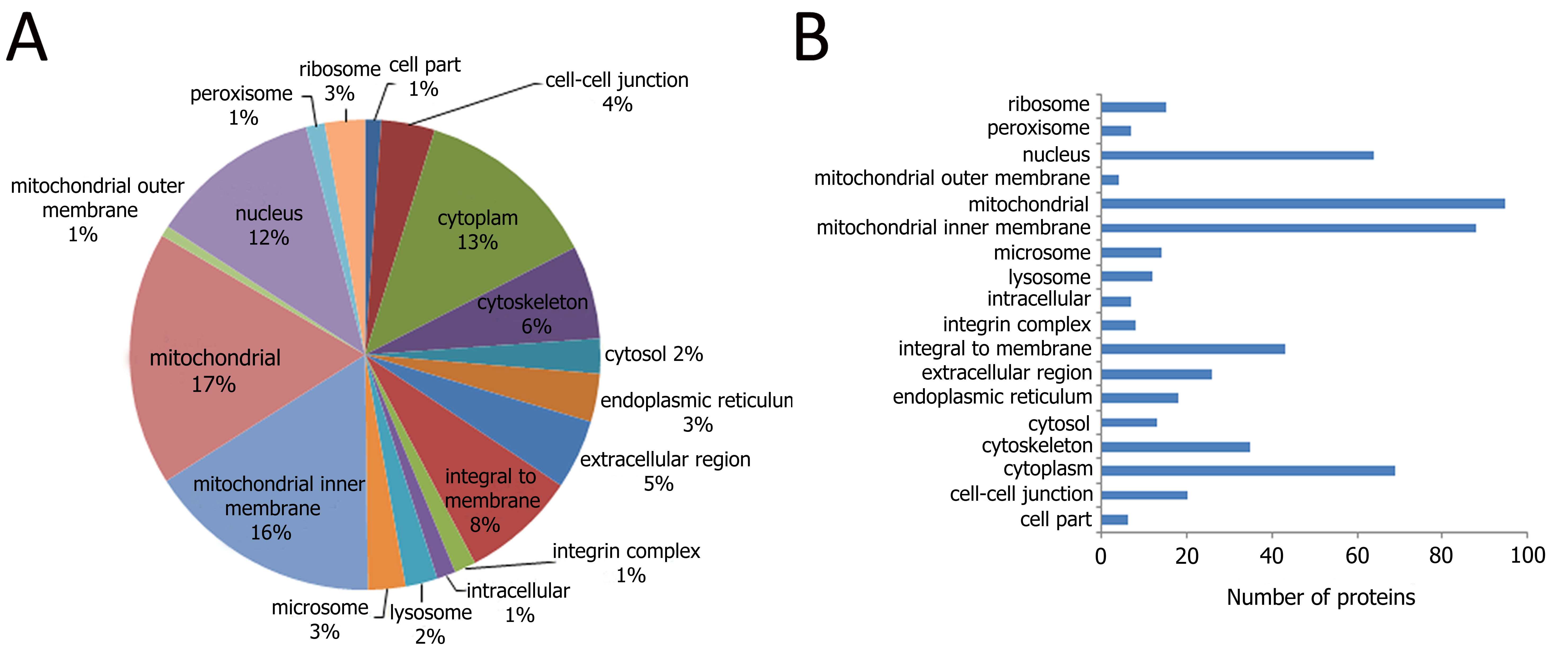

Supplement: Figure S2 — Isolated capillary protein classification according to cellular component. A, GO annotation by cellular component. 544 proteins were identified to be associated with a diverse cellular component and mitochondrial proteins, including matrix proteins (17%), inner membrane proteins (16%) and outer membrane proteins (1%), were highly expressed in the blood-labyrinth barrier. B, Allocation of isolated stria vascularis capillary proteins by cellular component showed the greatest number of these proteins (95) were allocated to a mitochondrial matrix. (TIF) [file pone.0016547.s002.tif]

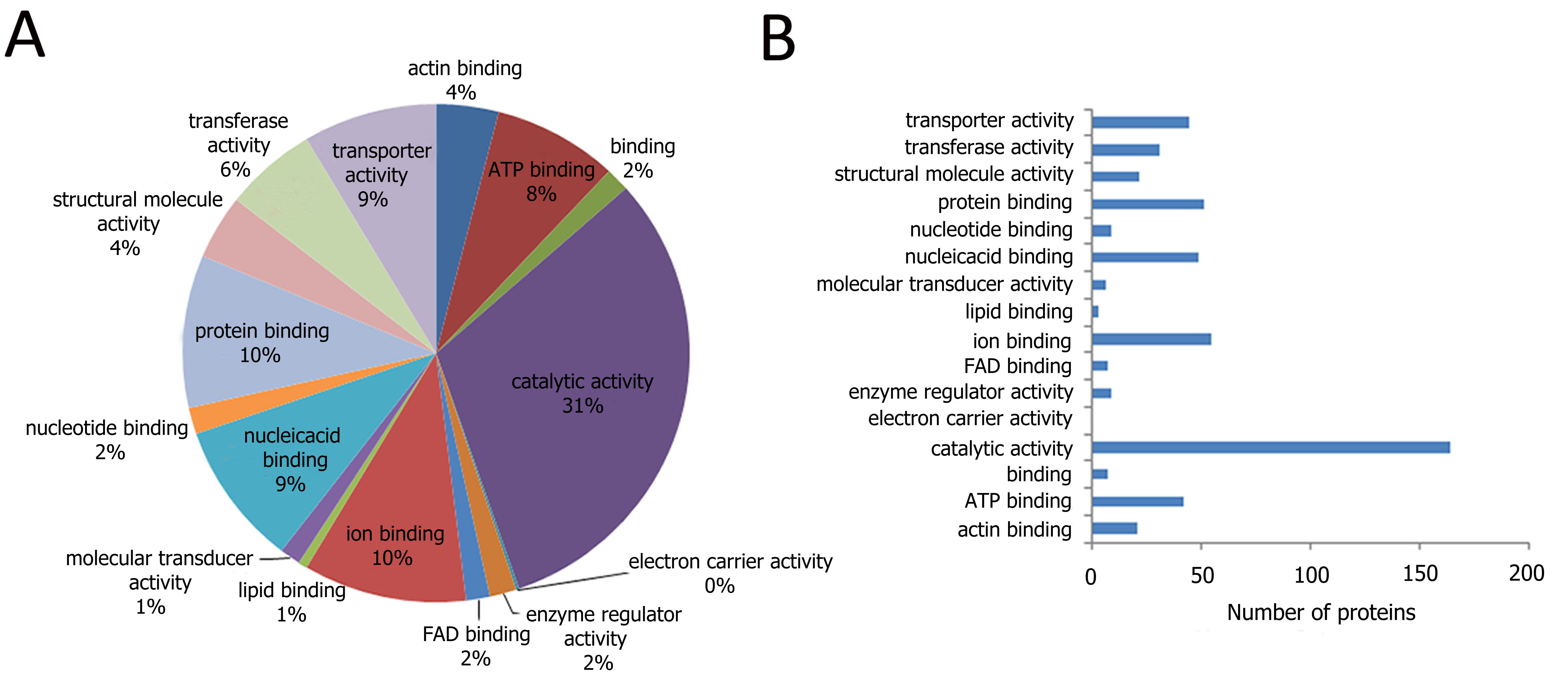

Supplement: Figure S3 — Isolated capillary protein classification according to molecular function. A, GO annotation by molecular function. 526 proteins were identified to be related to molecular functions and proteins involved in catalytic activity (31%) were highly expressed in the blood-labyrinth barrier. B, Allocation of isolated stria vascularis capillary proteins by molecular function showed that the greatest number of these proteins (164) was allocated to catalytic activity and the smallest (1) to electron carrier activity. (TIF) [file pone.0016547.s003.tif]

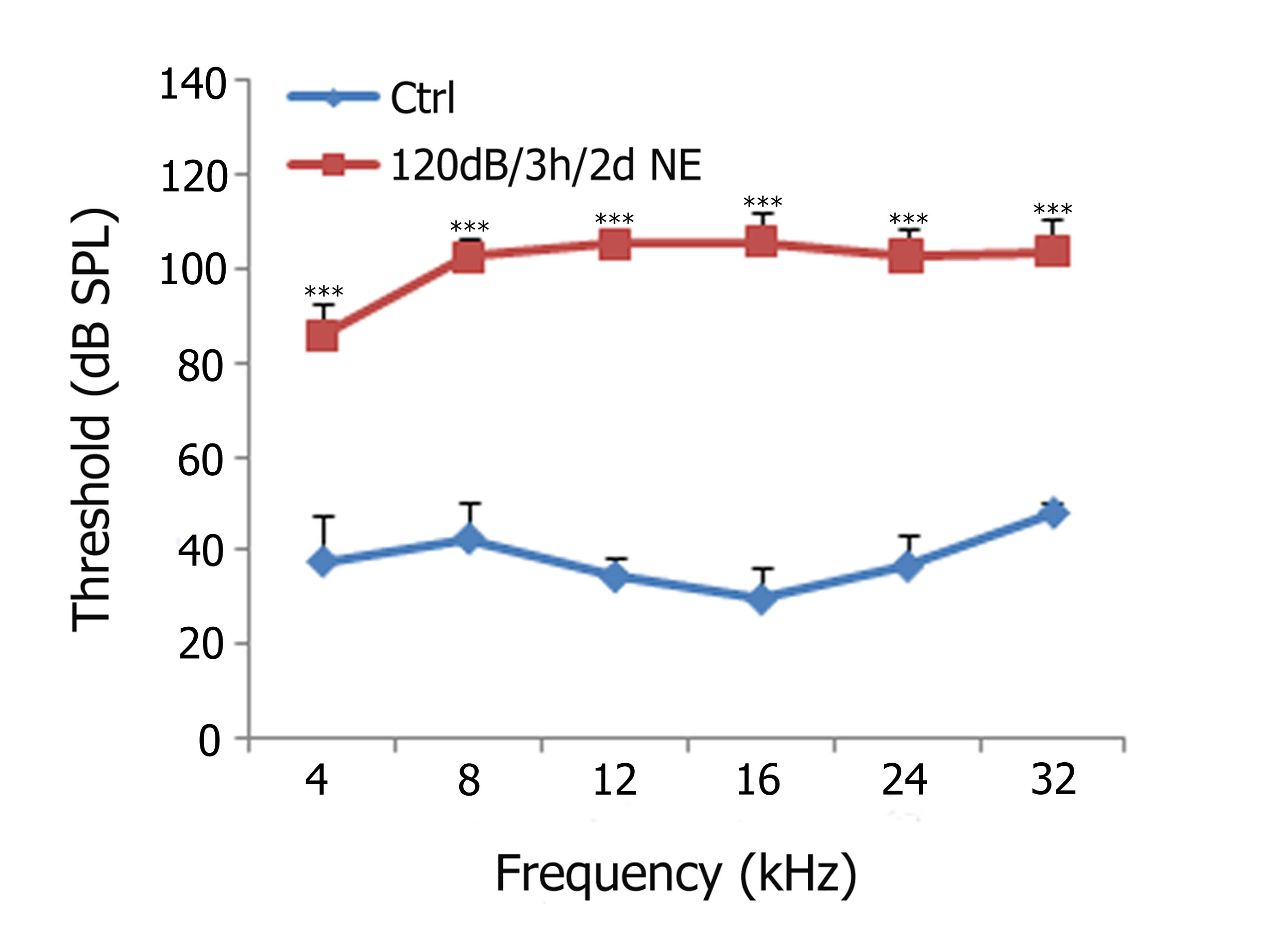

Supplement: Figure S4 — ABR hearing thresholds were measured from the noise-exposed and control mice (n = 10) at 4, 8, 12, 16, 24 and 32 kHz. *Significantly different from control mice (***P < 0.001). Error bars represent SEM. (TIF) [file pone.0016547.s004.tif]

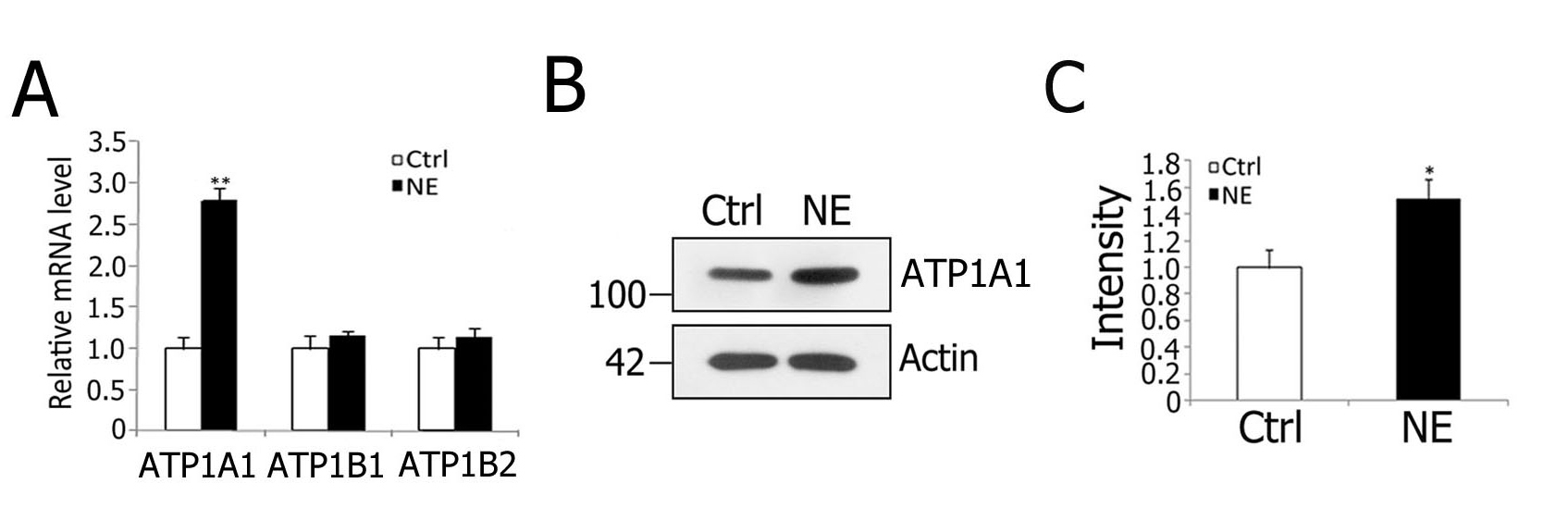

Supplement: Figure S5 — The expression of ATP1A1 in response to noise exposure. A, Real-time PCR showed the expression of mRNAs of Na+, ATP1A1, ATP1B1 and ATP1B2 in the control and noise-exposed (NE) animals. ATP1A1 mRNA was highly up-regulated in response to noise exposure. (** P = 0.0043< 0.01, n = 3). However, ATP1B1 and ATP1B2 mRNA did not reveal a significant difference in NE animals. B and C, Immunoblots quantified by densitometry shows that expression of ATP1A1 was significantly increased in the NE animals. (* P = 0.032 < 0.05, n = 5). (TIF) [file pone.0016547.s005.tif]
